# Protective effect of guggulsterone against cardiomyocyte injury induced by doxorubicin in vitro

**DOI:** 10.1186/1472-6882-12-138

**Published:** 2012-08-27

**Authors:** Wen-Ching Wang, Yih-Huei Uen, Ming-Long Chang, Khoot-Peng Cheah, Joe-Sharg Li, Wen-Yu Yu, Kock-Chee Lee, Cheuk-Sing Choy, Chien-Ming Hu

**Affiliations:** 1 Department of Surgery; Department of Medical Research, Chi Mei Medical Center, Tainan, Taiwan; 2 Department of Medical Research Chi Mei Medical Center, Tainan, Institute of Biomedical Engineering, Southern Taiwan University, Tainan, Taiwan; 3 Emergency Department, Taipei Medical University Hospital, Taipei, Taiwan; 4 Taipei Hospital, Department of Health, Taipei, Taiwan; 5 Department of Primary Care Medicine, School of Medicine, College of Medicine, Taipei Medical University; Emergency Department, Taipei Medical University Hospital, Wu-Xing Street, Taipei, 110, Taiwan

**Keywords:** Guggulsterone, Doxorubicin, Cardiotoxicity, Cytokines, Reactive oxygen species

## Abstract

**Background:**

Doxorubicin (DOX) is an effective antineoplastic drug; however, clinical use of DOX is limited by its dose-dependent cardiotoxicity. It is well known that reactive oxygen species (ROS) play a vital role in the pathological process of DOX-induced cardiotoxicity. For this study, we evaluated the protective effects of guggulsterone (GS), a steroid obtained from myrrh, to determine its preliminary mechanisms in defending against DOX-induced cytotoxicity in H9C2 cells.

**Methods:**

In this study, we used a 3-(4,5-dimethylthiazol-2-yl)-2,5-diphenyl-2H-tetrazolium bromide (MTT) assay, lactate dehydrogenase (LDH) release measurements, and Hoechst 33258 staining to evaluate the protective effect of GS against DOX-induced cytotoxicity in H9C2 cells. In addition, we observed the immunofluorescence of intracellular ROS and measured lipid peroxidation, caspase-3 activity, and apoptosis-related proteins by using Western blotting.

**Results:**

The MTT assay and LDH release showed that treatment using GS (1–30 μM) did not cause cytotoxicity. Furthermore, GS inhibited DOX (1 μM)-induced cytotoxicity in a concentration-dependent manner. Hoechst 33258 staining showed that GS significantly reduced DOX-induced apoptosis and cell death. Using GS at a dose of 10–30 μM significantly reduced intracellular ROS and the formation of MDA in the supernatant of DOX-treated H9C2 cells and suppressed caspase-3 activity to reference levels. In immunoblot analysis, pretreatment using GS significantly reversed DOX-induced decrease of PARP, caspase-3 and bcl-2, and increase of bax, cytochrome C release, cleaved-PARP and cleaved-caspase-3. In addition, the properties of DOX-induced cancer cell (DLD-1 cells) death did not interfere when combined GS and DOX.

**Conclusion:**

These data provide considerable evidence that GS could serve as a novel cardioprotective agent against DOX-induced cardiotoxicity.

## Background

Doxorubicin (DOX), a member of the anthracycline class of chemotherapeutic drugs, is a potent antineoplastic drug that has exhibited a wide spectrum of antitumor activity including in leukemia, lymphomas, soft tissue sarcomas, and breast cancer for several decades. Despite the efficacy of DOX, its use has been limited by the dose-dependent cardiotoxicity associated with acute and chronic treatments in anticancer therapy [1-3]. Both acute and chronic DOX-induced cardiotoxicity may cause cardiac dysfunction or cardiomyopathy, and may ultimately lead to rigorous heart failure and death [3,4]. Previous studies have suggested that one mechanism responsible for DOX cardiotoxicity is the formation of reactive oxygen species (ROS) [5,6], which can harm membrane lipids and other cellular components, leading to cardiomyocyte apoptosis and death [7]. Certain antioxidants have been examined to reduce ROS formation, but many have demonstrated only a limited cardioprotective effect or have other side effects [8,9].

**Figure 1:**
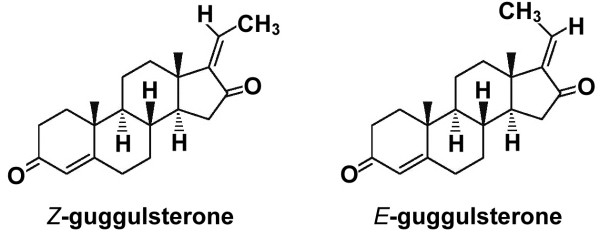
**Structure of*Z*-guggulsterone [*trans*-4,17(20)-pregnadiene-3,16-dione]**[12]**.**

Guggulsterone (GS) is a steroid compound that has been isolated from myrrh [10,11]; the structure of which is shown in Figure 1[12]. In 1972, two isomer derivatives, *Z*-GS and *E*-GS, were identified in *Commiphora mukul* (containing 4.50% (*Z*)-isomer and 1.42% (*E*)-isomer) [13] as antagonist ligands for the bile acid receptor farnesoid X receptor and as active ingredients responsible for hypolipidemic activity [14,15]. During a 10-year period, myrrh was examined to treat various conditions, such as rheumatism, atherosclerosis, hypercholesterolemia, and obesity [16]. Both GS isomers were demonstrated to suppress LPS-induced inflammation by inhibiting IκB-α degradation and NF-κB activation [17,18]. GS was mediated (possibly by activating protein-1) to upregulate the expression of the bile salt export pump [16]. In addition, GS was reported to protect cardiac and neuronal damages in animal models [19]. However, no research has yet been conducted on the use of GS in protecting against DOX-induced cardiotoxicity. This study is the first to evaluate the protective effects of GS to determine the preliminary mechanisms of defending against DOX-induced cytotoxicity in H9C2 cells.

## Methods

### Chemicals

Dulbecco’s Modified Eagle’s Medium, an RPMI 1640 medium, fetal bovine serum, penicillin/streptomycin, and medium supplements were purchased from Life Technologies (Gibco, Grand Island, NY). Monoclonal antibodies and a peroxidase-conjugated secondary antibody were purchased from Santa Cruz Biotechnology (Santa Cruz, CA). DOX, GS, and other agents were obtained from Sigma Chemical (St. Louis, MO).

### Cell culture

All rat cardiac H9C2 myocardial cells, spontaneously immortalized ventricular rat embryo myoblasts, and DLD-1 cells (human colon adenocarcinoma) were purchased from the Food Industry Research and Development Institute, Taiwan (BCRC). The H9C2 cells were cultured in DMEM supplemented with 10% fetal bovine serum at 37°C in 5% CO_2_, and DLD-1 cells were cultured in the RPMI 1640 medium. The media were changed every 2–3 d.

### Cell viability

Sub-confluent cells were trypsinized and seeded onto 96-well plates at a density of 1.5 × 10^5^ cells/ml and incubated for 24 h before treatment. Thereafter, the cells were exposed to DOX 1 μM for 24 h and then incubated in a fresh medium with *Z*-GS at various concentrations for an additional 24 h. The effects of GS on DOX-induced cytotoxicity were assessed using the MTT assay, as previously described [20]. The unwashed dye was eluted and quantified spectrophotometrically at 550 nm using a microplate reader. Cell viability was determined as the percentage of surviving cells compared with that of the DOX-treated control.

### Lactate dehydrogenase (LDH) release assay for cytotoxicity

GS-induced cytotoxicity leading to plasma membrane damage was measured using the LDH Cytotoxicity Detection Kit (Boehringer Mannheim, Mannheim, Germany). The LDH release assay has been widely used in cytotoxicity studies [21]. The detailed assay was performed as previously described [22].

### Intracellular ROS

Intracellular ROS induced by DOX was measured using 2’7’-dichlorodihydrofluorescein diacetate (DCFH-DA) as a fluorescent probe [23]. H9C2 cells were loaded with DCFH-DA (20 μ) for 10 min, followed by 2 washes with HBSS. Dichlorodihydrofluorescein (DCF) fluorescence was detected using a fluorescence spectrophotometer with an excitation of 485 nm and an emission of 520 nm, and the fluorescence image was visualized using a fluorescence microscope.

### Lipid peroxidation

Lipid peroxidation was assayed using the thiobarbituric acid (TBA) reaction. The amount of thiobarbituric acid reactive substance (TBARS) was determined using a standard curve of 1,1,3,3-tetramethoxypropane. The detailed assay procedure was performed as previously described [24].

### Preparation of total cell lysates and nuclear and cytosolic extracts

H9C2 cells (5 × 10^5^ cells/well) or DLD-1 cells in 6-well plates were incubated with or without concentrations of GS and DOX (1 μM) for 24 h. The total cell lysates were obtained using a lysis buffer (250 mM Tris–HCl (pH 6.8), 1% Triton-100, 0.1% SDS, 1 mM Na_3_VO_4_, 1 mM EDTA, 5 mM sodium fluoride, 1 mM PMSF, and 1 mg/ml leupeptin), and cell debris was removed using a centrifuge at 10 000 × *g* for 10 min at 4°C and stored at −80°C until required. The protein content of the cell lysates was determined using the Bradford assay [25].

### Western blot analysis

Equal amounts of cell lysates (30 μg) were electroblotted onto a nitrocellulose membrane (Millipore, MA), following separation using 8%-12% SDS-polyacrylamide gel electrophoresis. The blot was probed using a primary antibody against poly (ADP-ribose) polymerase (PARP), caspase-3, bcl-2, bax, cytochrome C, and β-actin (Santa Cruz Biochemicals, Santa Cruz, CA). The intensity of each band was quantified using density analysis software (MetaMorph Imaging System, Meta Imaging Series 4.5), and the density ratio represented the relative intensity of each band against controls in each experiment.

### Hoechst 33258 staining

Cells were rinsed twice in 4°C PBS and fixed in 4% formaldehyde at 4°C for 10 min. After washing, the cells were incubated using Hoechst 33258 (5 μg/ml) staining at room temperature for 10 min in the dark. The cells were then observed and imaged using a laser scanning confocal microscope (Bio-Rad MRC-1000, American Laboratory, USA) with an excitation of 350 nm and an emission of 460 nm [26].

### Caspase-3 activity assay

Caspase-3 activity was determined using the ApoAlert Caspase Colorimetric Assay kit (Clontech Laboratories, Inc. USA), according to manufacturer’s protocol.

### Data and statistical analysis

The results of all the experiments were expressed as the mean ± standard error (SE) obtained from the number of replicate treatments. Data were analyzed using analysis of variance (ANOVA), followed by Dunn’s post hoc test for comparison, and P values of < 0.05 were considered statistically significant.

## Results

### Effects of guggulsterone (GS) on cell viability and cytotoxicity

**Figure 2:**
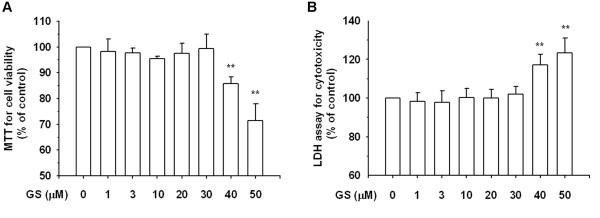
**Effects of guggulsterone (GS) on the H9C2 cell line.** (**A**) Cells were treated with various concentrations of GS for 24 h. Cell viability was determined using an MTT assay, as described in the Materials and Methods section. (**B**) GS cytotoxicity was analyzed using LDH release. The results are expressed as mean ± S.E. from 3 independent experiments. Asterisks indicate a significant difference for the control (GS 0 μM) (**P < 0.01).

The cell survival percentages of H9C2 cells receiving GS treatment were measured using an MTT assay and an LDH release assay. As shown in Figure 2A and 2B, the cells were not significantly injured by GS treatment at up to 30 μM. These results suggest that GS concentrations were nontoxic to H9C2 cells below 30 μM. Thus, the cells were treated with GS in concentrations ranging from 1 to 30 μM for all follow-up experiments.

### GS protected H9C2 cells from DOX-induced cell death

**Figure 3:**
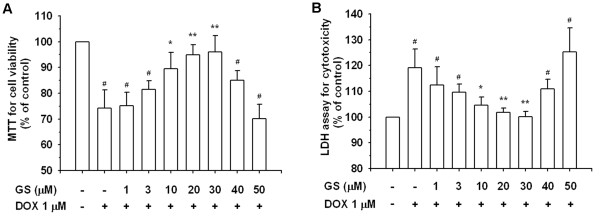
**GS prevention on DOX-induced H9C2 cell death.** Cells were incubated with DOX (1 μM) in the presence and absence of GS for 24 h. The percentages of viable cells were determined using an MTT assay, as described in the Materials and Methods section. The results are expressed as mean±S.E. from 3 independent experiments. ^*^P < 0.05 and ^**^P < 0.01 indicate a significant difference from DOX treatment. ^*#*^P < 0.01 indicates a significant difference from the control (GS 0 μM and DOX 0 μM).

**Figure 4:**
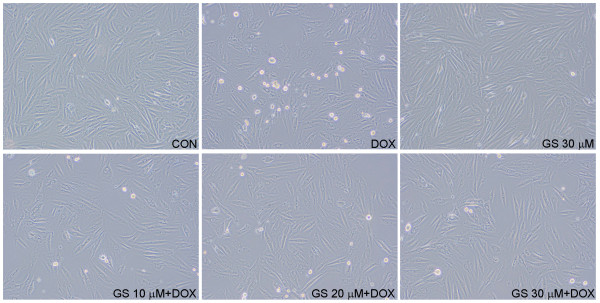
**Morphology of DOX-induced cell death in H9C2 cells in the presence or absence of GS for 24 h.** Cells were viewed using a video camera (Nikon) attached to a microscope, and the image was projected onto a monitor. Images were obtained with a 200× objective.

To determine GS concentrations prevented in DOX-induced cytotoxicity, we examined the preventive abilities of GS in H9C2 cells at different concentrations. The results of the MTT assay and LDH release assay showed that exposure to DOX at a concentration of 1 μM for 24 h caused 25.9% cell death (Figure 3A); however, no significant reduction of viable cells was found in 10–30 μM GS-treated cells after 24 h treatment (Figure 3A and 3B). These findings show that GS at 10–30 μM can reduce DOX-induced cytotoxicity in H9C2 cells. Microscopic examinations of the cell cultures showed a reversal of DOX-induced cell death in cell morphology when treated with differing concentrations of GS (10, 20, and 30 μM), as shown in Figure 4.

### GS relieved oxidative stress induced by DOX in H9C2 cells

**Figure 5:**
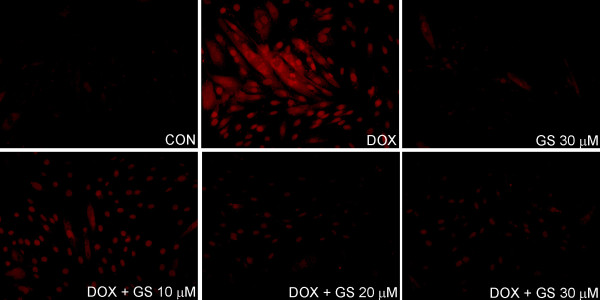
**GS relieved the oxidative stress induced by DOX in H9C2 cells.** Staining of intracellular ROS by dihydroethidium (DHE) staining in H9C2 cells (original magnification × 200).

**Figure 6:**
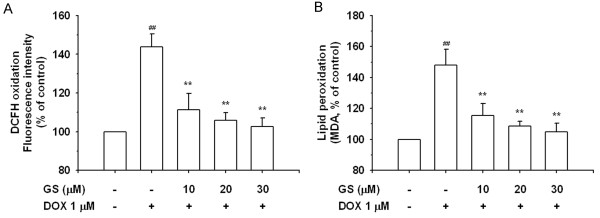
**Prevention of GS on DOX-induced oxidative stress.** Subconfluent H9C2 cells were left untreated or exposed to DOX and co-treated with GS for 24 h and oxidative stress was evaluated by measuring DCFH oxidation (**A**) and the amount of TBARS formation (malondialdehyde, MAD) (**B**). The results are expressed as mean±S.E. from 3 independent experiments. ^**^P < 0.01 indicates a significant difference from DOX treatment. ^*##*^P < 0.01 indicates a significant difference from the control (GS 0 μM and DOX 0 μM).

As shown in Figure 5, cells exposed to DOX were significantly brighter than the control cells with numerous highlights, where ROS was concentrated in dihydroethidium (DHE) staining. By contrast, the red fluorescence intensity of the cells pretreated with GS was considerably darker than or nearly as dark as the control cells. Moreover, the ROS level of the cells treated with GS alone was nearly the same as that of the control cells. Nevertheless, we measured the oxidation of DCFH and lipid peroxidation, which are widely used markers of intracellular ROS formation, in H9C2 cells treated for 24 h with 1 μM DOX in the presence or absence of GS, as shown in Figure 6A and 6B.

### GS protected against apoptosis of H9C2 cells treated with DOX

**Figure 7:**
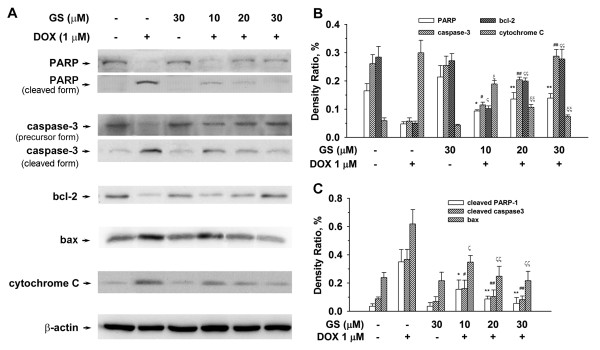
**Effects of GS on DOX-induced apoptosis-related protein in the cytoplasm of H9C2 cells.** (**A**) Cells (5 × 10^5^ cells/ well) were pretreated with the indicated concentrations of GS for 2 h before incubation with DOX (1 μM) for 24 h. The proteins (30 μg) of total cell lysates were analyzed at the expression levels of anti-PARP, caspase-3, bcl-2, bax, and cytosolic lysates that were analyzed for anti-cytochrome C by Western blotting at 10% SDS-PAGE. β-Actin is the internal standard to confirm equal loading. (**B**) Quantification of PARP, bcl-2, and caspase-3 expression, and cytochrome C release. (**C**) Quantification of the cleaved form of PARP and caspase-3, as well as the bax expression. Data are expressed as mean ± S.E. from 3 independent experiments. ^*^P < 0.05, ^**^P < 0.01, and PARP (both the precursor and cleaved form) expression compared to the DOX treatment alone; ^#^P < 0.05 and ^##^P < 0.01, and caspase-3 (both the precursor and cleaved form) compared to the DOX treatment alone; ^ς^P < 0.05 and ^ςς^P < 0.01, and bcl-2 and bax compared to the DOX treatment alone. ^ξ^P < 0.05 and ^ξξ^P < 0.01, and cytochrome C compared to the DOX treatment alone.

**Figure 8:**
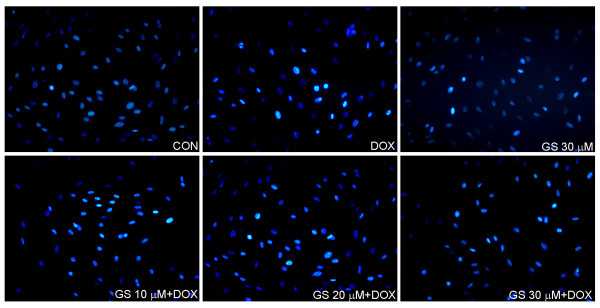
**The protective effects of GS on H9C2 cells against DOX-induced apoptosis by Hoechst 33258 staining.** Fluorescence images of Hoechst 33258 stained H9C2 cells after a 2 h of exposure to DOX in the absence or presence of GS. Images were obtained using a 200× objective.

**Figure 9:**
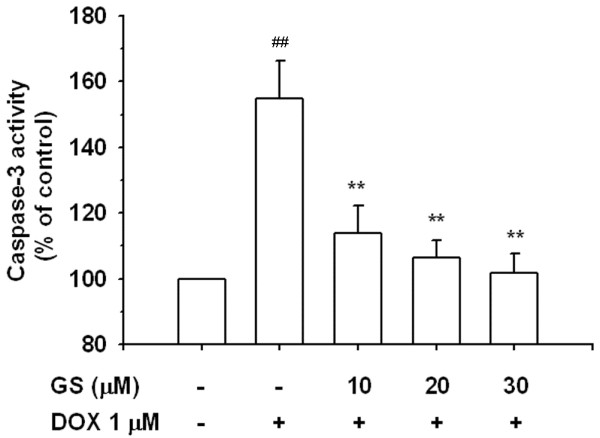
**Effects of GS on DOX-induced apoptosis using caspase-3 activity assay in H9C2 cells.** Subconfluent H9C2 cells were treated with DOX and co-treated with different concentrations of GS for 24 h, and caspase-3 activity was measured using colorimetry. The results are expressed as mean±S.E. from 3 independent experiments. ^**^P < 0.01 indicates a significant difference from DOX treatment. ^*##*^P < 0.01 indicates a significant difference from the control (GS 0 μM and DOX 0 μM).

We investigated the effects of GS on DOX-induced apoptosis in H9C2 cells by using Western blotting. PARP, caspase-3, and bcl-2 are major types of regulatory proteins associated with apoptosis. As shown in Figure 7A, PARP and bcl-2, two apoptotic suppressors, were markedly decreased and the cleaved PARP had increased after treatment with DOX (1 μM) for 24 h. By contrast, caspase-3 (both the precursor and cleaved form), a pro-apoptotic protein, was markedly altered after DOX treatment. However, these effects of DOX-induced PARP, caspase-3, and bcl-2 were reversed in a concentration-dependent manner by pretreating with GS for 2 h. In addition, bax and cytochrome C release decreased significantly following GS treatment. As shown in Figure 7B and 7C, the density ratio of PARP (both the precursor and cleaved form), caspase-3 (both the precursor and cleaved form), bcl-2, bax, and cytochrome C is represented in quantitative determination. The protective effects of GS on H9C2 cells were detected using the Hoechst 33258 stained and caspase-3 activity assays. As shown in Figure 8, normal cells were observed as round-shaped nuclei with homogeneous fluorescence intensity. DOX induced rapid nuclear changes of H9C2 with heterogeneous intensity and chromatin condensation. In the control and GS groups, most nuclei exhibited regular contours and were round and large. The GS + DOX group showed slight DNA condensation but only a few fragmentations of chromatin. This suggests that apoptosis was the prevalent form of cell death in H9C2 cells exposed to 1 μM of DOX. In addition, we further measured the activity of caspase-3, a major effector protein of apoptosis. As shown in Figure 9, caspase-3 activity was increased by 1 μM of DOX, and GS can inhibit DOX-induced caspase-3 activity at 10–30 μM after 24 h of treatment.

### GS did not interfere with DOX-induced cell death in DLD-1 cells

**Figure 10:**
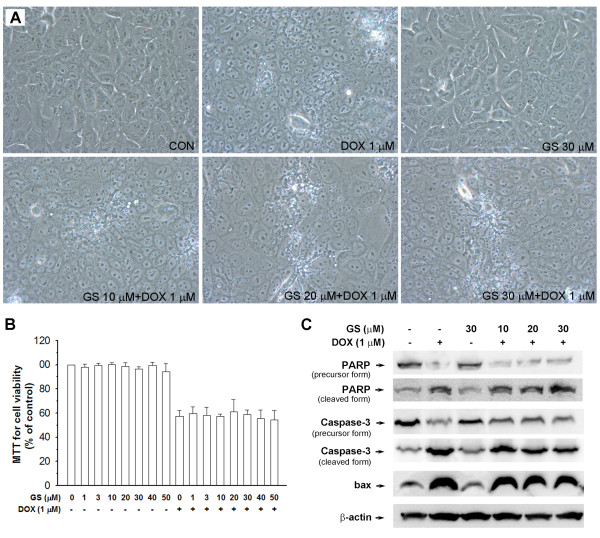
**Effects of combining GS and DOX in cancer cells.** (**A**) Morphology of DOX-induced cell death. (**B**) Cell viability of the MTT assay. (**C**) Western blotting of PARP, caspase-3 and bax protein expression in DLD-1 cells in the presence or absence of GS for 24 h. Images were obtained with a 200× objective of the microscope. The assay methods were performed in the same manner as that described for the H9C2 cells.

To determine whether GS influences DOX in cancer therapy, GS and DOX were combined to treat DLD-1 cells (human colon adenocarcinoma). As shown in Figure 10A, the morphology of DLD-1 cells indicated that they were injured after treatment with 1 μM of DOX; however, GS (10, 20 and 30μM) did not reverse this response. As shown in Figure 10B, GS (1–50 μM) did not cause cytotoxicity in the DLD-1 cells. DOX (1 μM) can induce 42.6% cell death; however, the cell viability did not significantly increase after co-treatment of GS (1–50 μM) and DOX. In addition, we assessed PARP, caspase-3, and bax by Western blotting. As shown in Figure 10C, DOX induced PARP and caspase-3 protein degradation, increased cleaved-PARP and cleaved-caspase-3 expression, and induced bax expression. However, these effects were not influenced by co-treatment with GS.

## Discussion

DOX, as an anthracycline antibiotic, has been used in cancer therapy for nearly three decades. However, the clinical use of DOX is restricted considerably by an increased risk of cardiotoxicity associated with DOX-induced cardiomyocyte apoptosis [27-29]. Adult cardiomyocytes of the heart are well known to finally differentiated muscle cells that do not progress proliferation [30]. In addition, DOX seemed to affect specific enzymes (NADH and FADH_2_), transporters (Ca^2+^-ATPase and Na^+^, and K^+^-ATPase), and metabolic pathways (AMP-activated protein kinase) to varying extents in the cardiac muscle. The accumulation of these defects may ultimately result in irreversible cardiac failure [31]. This study elucidated the protection of GS from DOX-induced apoptosis and cell death by conducting the following experiments: MTT assay, LDH release, DHE-staining, fluorescence intensity of DCFH, lipid peroxidation, Hoechst 33258 staining, caspase-3 activity measurement and immunoblot analysis. The production of ROS as a derivative of the DOX metabolism has been suggested to be the main mechanism of DOX-induced cardiotoxicity [32-34]. However, superoxide radicals are involved in other ROS and generate hydroxyl radical and hydrogen peroxide [35]. According to current reports, generating these ROS causes mitochondrial damage, which may lead to cardiomyocyte apoptosis, necrosis, or death. In addition, these DOX-induced mitochondrial injuries in cardiomyocytes were prevented by some natural substances, such as curcumin, naringenin-7-O-glucoside, and plantainoside D [20,36,37]. In our study, we used DCFH-DA as an intracellular method of ROS detection [38]. The results demonstrated that GS can significantly reduce the intracellular ROS produced by DOX in H9C2 cells. We also measured lipid peroxidation (MDA formation) in H9C2 cells. The reduction of MDA content suggested that GS can attenuate the oxidative stress induced by DOX in H9C2 cells.

According to the identified apoptosis of cell signaling, PARP is a nuclear enzyme activated by strand breaks in DNA and implicated in DNA repair, apoptosis, organ dysfunction, or necrosis [39,40]. In addition, it is known that members of the bcl-2 protein family are known to be major regulators of cytochrome C release and downstream caspases activation. Thus, bcl-2 plays a vital role in regulating cardiomyocyte apoptosis [29,41]. Using Western blot analysis, we found that GS treatment attenuated DOX-induced apoptotic proteins (cleaved-PARP, cleaved-caspase-3, and bax). In contrast, treatment with GS also enhanced anti-apoptotic protein (bcl-2) expression in cardiomyocytes. Finally, another type of cancer cell line, DLD-1, was used to exclude the possible interference of GS with DOX in cancer therapy. The data showed that the efficacy of DOX did not diminish co-treatment of GS and DOX.

## Conclusion

Numerous studies have shown that myocardial impairment caused by DOX may be due to cardiomyocyte apoptosis, and DOX may also cause injury to endothelial cells [42]. This study shows that GS is a novel potent protector against DOX-induced cardiotoxicity, providing protection using its antioxidative activity.

## Competing interests

The authors declare that they have no competing interests.

## Authors’ contributions

WCW and YHU contributed equally to this work. WCW, YHU and CMH participated in the design and coordination of the study, carried out the analyses and wrote the manuscript. MLC, KPC and JSL helped to operate the confocal microscope and helped to draft the manuscript. WYY, KCL and CSC helped to analyze data for statistical analysis. CMH participated in the data interpretation and manuscript preparation. All authors read and approved the final manuscript.

## Pre-publication history

The pre-publication history for this paper can be accessed here:

http://www.biomedcentral.com/1472-6882/12/138/prepub
